# Contributions of global and local processing on medical image perception

**DOI:** 10.1117/1.JMI.10.S1.S11911

**Published:** 2023-05-08

**Authors:** Hyung-Bum Park, Lilian Azer, Shinhae Ahn, Tam-Dan Dinh, Gabriela Macias, Gavin Zhang, Bihong Beth Chen, Huiyan Ma, Mahesh Botejue, Eric H. Choi, Weiwei Zhang

**Affiliations:** a The University of Chicago, Institute for Mind and Biology, Chicago, Illinois, United States; b University of California, Riverside, Department of Psychology, Riverside, California, United States; c Washington University in St. Louis, Department of Psychological and Brain Sciences, St. Louis, Missouri, United States; d University of California, Berkeley, Department of Computer Science, Berkeley, California, United States; e City of Hope National Medical Center, Department of Diagnostic Radiology, Duarte, California, United States; f City of Hope National Medical Center, Beckman Research Institute, Department of Population Sciences, Duarte, California, United States; g University of California Riverside School of Medicine, Riverside Community Hospital, Internal Medicine, Riverside, California, United States

**Keywords:** signal detection, mammography, holistic process, focal process, hierarchical Bayesian, mouse trajectory

## Abstract

**Purpose:**

The influential holistic processing hypothesis attributes expertise in medical image perception to cognitive processing of global gist information. However, it has remained unclear whether or how experts use rapid global impression of images for their subsequent diagnostic decisions based on the focal sign of cancer. We hypothesized that continuous-global and discrete-local processes jointly attribute to radiological experts’ detection of mammogram, with different weights and temporal dynamics.

**Approach:**

We examined experienced versus inexperienced observers’ performance at first (500 ms) versus second (2500 ms) mammogram image presentation in an abnormality detection task. We applied a dual-trace signal detection (DTSD) model of receiver operating characteristic (ROC) to assess the time-varying contributions of global and focal cancer signals on mammogram reading and medical expertise.

**Results:**

The hierarchical Bayesian DTSD modeling of empirical ROCs revealed that mammogram expertise (experienced versus inexperienced observers) manifests largely in a continuous-global component for the detection of the gist of abnormality at the early phase of mammogram reading. For the second presentation of the same mammogram images, the experienced participants showed increased task performance that was largely driven by better processing of discrete-local information, whereas the global processing of abnormality remained saturated from the first exposure. Modeling of the mouse trajectory of the confidence rating responses further revealed the temporal dynamics of global and focal processing.

**Conclusions:**

These results suggest a joint contribution of continuous-global and discrete-local processes on medical expertise, and these processes could be analytically dissociated.

## 1 Introduction

### 1.1 Global and Local Processes in Medical Image Perception

In natural vision, visual attributes arise not only from isolated objects (e.g., trees) but also from global and holistic representations (e.g., forest), often defined as “gist” or “emergent feature.”^1^ These global features are potentially important for forming the structure and regularities of the natural environment,^2^^,^^3^ and subsequently leading to natural scene understanding^4^ and object recognition and memory.^5^^–^^7^ For instance, higher-level holistic representations could provide some coarse-grained information (e.g., “what is it like”)^8^ to guide or constrain the processing of discrete-local information (e.g., “what is it”).^8^ Extraction of the gist from the visual scene may involve a rapid, nonselective process with minimal involvement of controlled cognitive processes, such as attention and consciousness.^2^^,^^9^

In the domain of medical image perception, global visual processing is thought to enable radiologists to sense the possible abnormality in medical images such as mammograms before localizing the focal signs of the abnormality (e.g., malignant lesions).^10^^,^^11^ The influential holistic processing account^11^^–^^14^ attributes medical expertise to the cognitive processing of holistic and gist information, which is likely supported by the fusiform face area (FFA),^15^^–^^19^ a dedicated brain region for visual expertise.^20^ Mounting evidence has shown that experienced radiologists could rapidly extract diagnostic information and determine an abnormality of a medical image within 200 to 500 ms,^10^^,^^21^ manifesting as above chance performance in an abnormality detection task (e.g., signal detection $d^{\prime }>1.0$).^22^^–^^25^ Furthermore, expert radiologists can detect the presence of abnormality from a glimpse, even before any visible signs of abnormality have developed,^10^^,^^11^ suggesting that the gist of abnormality is global and perceptual in nature.

Nonetheless, the holistic processing account is at odds with some empirical findings for the involvement of local target processing^19^^,^^26^^,^^27^ and radiologists’ first-hand experience when reading mammogram images (e.g., they can often locate the focal sign of abnormality). In addition, it has remained unclear whether or how experts use rapid global impression of images for their subsequent diagnostic decisions based on the focal sign of cancer. Previous research largely focused on isolating the holistic processing of mammogram images using specific experimental procedures, such as parafoveal or rapid presentation of mammogram images.^26^^–^^28^ For example, a rapid flash of a medical image for about 200 to 500 ms gives the participants only a glimpse of the image while limiting volitional eye movements toward and thus attentive examinations of local parts of the image. These artificial procedures make it difficult to understand how radiologists read mammograms in typical clinical settings.

Earlier theoretical frameworks, including the two-stage detection models^29^ and the global-focal search model,^30^ proposed two visual processes that are qualitatively as well as temporarily dissociable with each other. The first stage rapidly gains global information about the image, whereas the second stage involves the subsequent search for a set of candidate regions of the image in a discrete manner. Although these models to some extent vary in their conceptualizations of holistic/global gist information (e.g., the content of initial global image perception), they offer a similar mechanism for the interactions between the two processes. Specifically, experts use their initial impression of an image to guide their subsequent search, consistent with the vast literature on visual search.^14^^,^^31^^,^^32^

Expertise in grasping the gist of abnormality, although useful, does not always lead to a correct diagnostic decision. Previous studies have shown that performance can increase substantially over image exposure duration, such as from $d^{\prime }>1.0$ for 250 or 500 ms (Ref. 21) to $d^{\prime }>2.0$ for free-viewing conditions.^24^ Even upon a successful detection of an abnormality, experts often fail to localize the abnormal lesion under the brief image exposure condition.^21^ A recent study using a gaze-contingent flash-preview moving window paradigm^26^ reported no diagnostical utility of 250 ms brief preview for the subsequent search for abnormality in the same image. Together, these raise a theoretically important question on the functional limit of the initial holistic processing. To address this question, it is pivotal to precisely characterize these seemingly distinct operations of global and local processing in medical image reading.

### 1.2 This Study

**Fig. 1:**
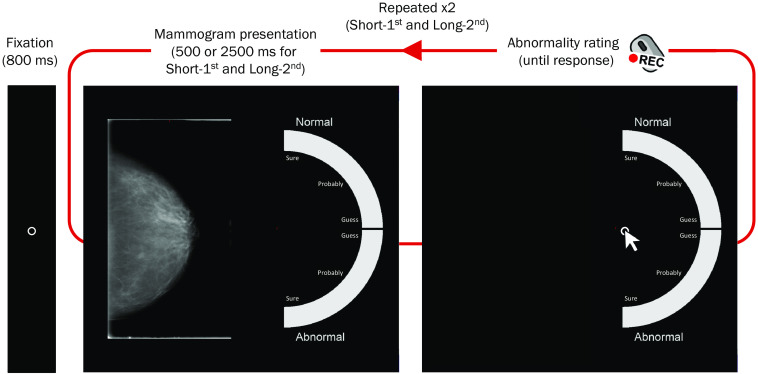
Mammogram abnormality detection task. Each trial began with an 800-ms central fixation, followed by the first mammogram image presentation at the center of the screen for 500 ms (short-first). After image offset, participants reported whether the image is normal or abnormal along with the level of confidence by clicking the mouse cursor on the continuous confidence scale presented on the right side of the screen. Mouse trajectory was recorded. After the first response, the same image was presented for 2500 ms (long-second) and participants again reported the abnormality and confidence.

This study has taken a model-based approach to quantitatively assess the time-varying contributions of global and focal cancer signals in mammogram reading and medical expertise. It is hypothesized that two dissociable cognitive processes (“discrete-local” and “continuous-global”) jointly support mammogram diagnostics but with different time courses. To test this idea, we adopted a mammogram abnormality detection task^10^ (Fig. 1). On each trial, the participants viewed a mammogram image twice, for 500 and 2500 ms in sequence. For each image presentation, they reported whether the image was normal verses abnormal as well as the level of confidence, by clicking the mouse cursor on a continuous scale. These responses were converted to receiver operating characteristic (ROC) curves, plotting true-positive (i.e., “abnormal” response for malignant image) rates against false-positive (i.e., “abnormal” response for benign image) rates along the variable response confidence, and then fit with a model (see next paragraph) to yield quantitative measures of local and global processes. The shape of ROC curves entail critical features for distinguishing cognitive processes.^33^^,^^34^ In addition, participants’ mouse trajectories from the center of the response scale to the landing point of mouse clicking were recorded to estimate the moment-by-moment influences of the dissociable perceptual processing of the mammogram image.

**Fig. 2:**
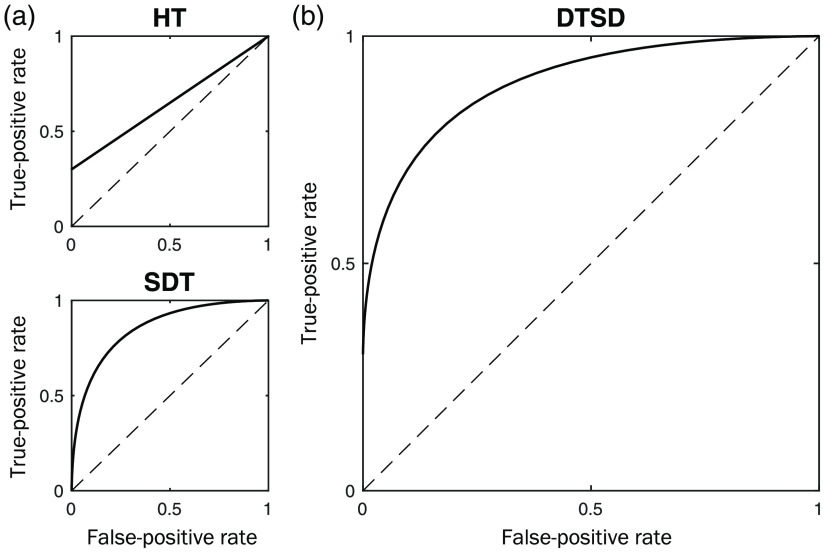
DTSD model. (a) The main idea of the DTSD model is that the curvilinear and asymmetrical ROCs (b) observed from diagnostic medical decisions can be modeled as a mixture of two distinct components: (1) high-threshold component (HT, top-left) for the discrete-local processing of focal signs of abnormality (e.g., identification of malignant lesions) manifests as linear ROCs. (2) SDT component ($d^{\prime }$, bottom-left) for the continuous-global processing of the gist of abnormality (e.g., observer’s prior knowledge about the visual patterns of abnormal images) manifest as symmetrical and curvilinear ROCs.

To measure the two dissociable processes, we adopted the dual-trace signal detection (DTSD) model (Fig. 2), developed from previous cognitive modeling of visual expertise.^5^^,^^35^ This model independently estimates global and local visual processes by combining two classes of ROCs with distinct shapes of ROC curve. Specifically, it conceptualizes global visual processing as a continuous process with graded strength that can be captured by a signal detection theory (SDT) component, global ($d^{\prime }$), of curvilinear ROCs (Fig. 2 bottom-left). In contrast, local processing is all or none, featuring linear ROCs with varying intercept at zero false-positives (Fig. 2 top-left), captured by a high-threshold component, local (HT). The DTSD can, therefore, model the empirical mammogram reading ROCs as the summation of global and local information processing [and hence curvilinear (SDT) and linear (HT) ROC functions] (Fig. 2 right).

The psychological validity of the DTSD model in dissociating and assessing global versus local information processing was established in the previous studies.^5^^,^^6^ First, the global ($d^{\prime }$) and the local (HT) components selectively respond to experimental manipulations of global and local information, respectively.^5^ Second, the global ($d^{\prime }$) component can predict global configural processing of face stimuli using an individual-difference approach.^6^ Note that the DTSD model is analytically equivalent to and in line with the recent adoption of the dual-process signal detection (DPSD) models for recognition memory^36^^,^^37^ to account for unconscious and automatic “sensing” versus controlled and conscious “perceiving” in perception and memory.^38^^,^^39^

Applying the DTSD model to mammogram abnormality detection, the continuous-global processing of the gist of abnormality can manifest as a curvilinear global ($d^{\prime }$) component, driven by expert’s rapid extraction of the global gist of an image and subsequent comparison to the prior knowledge about the visual patterns of abnormal images (e.g., mass and calcification). In contrast, the discrete-local processing of focal signs of mammogram abnormality (i.e., identification of malignant lesions and rejection of benign lesions) can manifest as a linear high-threshold component, local (HT). Note that although the overall abnormality detection performance (i.e., overall ROCs) is jointly determined by processing of global and local information (see Fig. 2), the two processes can be isolated and estimated using model fitting procedures.^5^ This is an approach widely used in the literature to distinguish and measure cognitive processes by fitting theoretical models to empirical ROC data^33^^,^^34^ (see Sec. 2.3.1 for detail).

The DTSD model makes distinct predictions for mammogram abnormality detection task performance in relation to image exposure duration (i.e., 500 versus 2500 ms) and the differences in radiological experience (i.e., experienced versus inexperienced observers). Specifically, for the first report after brief image exposure, medical expertise should primarily manifest as the global processing of abnormality, leading to increased strength modeled as the SDT component global ($d^{\prime }$) from inexperienced observers to experienced observers. This brief exposure may not be sufficient for the threshold-like discrete-local process^40^ to identify and locate the malignant lesion, rendering little effect of expertise on the local (HT) component. When the same image was presented again for a longer duration, the experts’ initial gist of abnormality will guide their examination and detection of local lesion. As a consequence, the guided search increases the likelihood of identifying a focal sign of malignancy, which will be captured by the local (HT) component of ROC.

## 2 Method

### 2.1 Participants

We recruited 26 conference attendees (10 female; average age 31.9 years) during the 2022 European Congress of Radiology as part of the National Cancer Institute Medical Image Perception Lab. All participants reported normal color vision and normal or corrected-to-normal visual acuity and were entered into a lottery to win a $50 or $25 Amazon gift card for their participation. For the grouping of radiological experience, 13 participants who are in a professional job position where they read medical images (e.g., radiologist, radiographer, radiology practitioner, physician, and physicist) with at least 3 years of experience were categorized as the “experienced” group (average age 34.9 years; average 7.8 years of experience; average about 2400 estimates of mammogram cases read per year), whereas the remaining 13 participants with little to no radiology experience (e.g., medical students, graduate students in radiological science, marketing, and territory manager) were categorized as “inexperienced” group (average age 28.9 years, average 1.4 years of radiological experience; average about 11 estimates of mammogram cases read per year). The experiment had Institutional Review Board approvals from the University of California, Riverside. Statistical power is evaluated from a data simulation and parameter recovery test of the hierarchical Bayesian DTSD model (see Sec. 2.3.1 for detail).

### 2.2 Stimuli and Procedure

Participants performed a mammogram abnormality detection task, in which a series of mammogram images, selected from the Curated Breast Imaging Subset of Digital Database for Screening Mammography (CBIS-DDSM) that includes the grayscale images of whole breast mammograms in standard Digital Imaging Communications in Medicine format, in craniocaudal or mediolateral oblique view, labeled with calcification or mass, and pathologically validated category as benign or malignant.^41^ Stimuli were presented on a 16-in. MacBook Pro laptop screen with a black background. Participants were instructed to maintain a viewing distance of around 60 cm.

Figure 1 illustrates the stimuli and procedure of the mammogram abnormality detection task. Each trial began with an 800 ms central fixation (0.2 deg in visual angle; 21 pixels), followed by the first presentation of a mammogram image (8.2  deg×8.2  deg; 860×860  pixels) at the display center for 500 ms and a half-circle confidence rating scale (radius of 8.2 deg or 860 pixels, thickness of 2.2 deg or 231 pixels) on the right side of the screen. After the mammogram image was off, the confidence scale remained on the display until the response. Specifically, participants reported whether the mammogram image is normal or abnormal, along with the level of confidence, by moving the mouse cursor from the fixed starting point (the center of the half circle scale) to a point on the confidence scale. The scale comprised of 90 points (1 to 90 from top to bottom) with the top (1) to the right-middle (45) of the half-circle scale representing continuous levels of confidence from sure, probably, to guess for “normal” responses, and the right-middle (46) to the bottom (90) of the scale representing the confidence levels from guess, probably, to sure for abnormal responses (labeled by text along the scale). [Note that 1 to 90 point scale was chosen for the convenience in converting the locations of points on a half-circle confidence rating scale to angular positions from the center of the scale (e.g., 0 to 90 points to 0 deg to 180 deg) for mouse trajectory data analysis with the circular statistics (see Sec. 2.3.3 for detail).] The choice of the response was confirmed with a mouse clicking and then marked with a cross for 500 ms. The cursor position was immediately set back to the confidence scale center.

The first reading response was followed by the second reading. The same mammogram image was presented again with the same response procedure, except that the image was presented for 2500 ms. The previous response on the confidence scale was unmarked prior to the beginning of the second reading to avoid a potential anchoring effect for the second response and participants were instructed that they can change their assessments between readings as much as needed. Participants’ mouse trajectory for each response was recorded. Half of the mammogram images were normal (i.e., images categorized as benign in the CBIS-DDSM), whereas the other half contained cancerous abnormalities (i.e., categorized as malignant). Both normal and abnormal images contained the same number of calcification and mass categories. Each participant performed a total of 40 trials (20 normal and 20 abnormal trials, randomly intermixed across trials). As our participant samples were not predetermined by their radiological experience in advance of recruitment, we provided all participants a short instruction of the task and target information (e.g., whether the image might contain cancerous lesions or otherwise might be something worthy of follow-up) with an example of abnormal image, and 10 practice trials at the beginning of the session. The overall procedure took about 15 min.

### 2.3 Data Analysis

#### 2.3.1 Hierarchical Bayesian DTSD modeling of ROC

ROCs were constructed from the confidence data for each participant at each response (first short- versus second long-image presentation). The 90-point confidence rating responses were binned into 20 bins, yielding 19 points on the mammogram reading ROC curve comprised of false-positive (abnormal response for normal trials) rates on the x axis and true-positive (abnormal response for abnormal trials) rates on the y axis. Note that abnormality was defined as the signal for SDT analyses.

The empirical ROCs was fit with the DTSD model using a hierarchical Bayesian method (HBM), instead of the traditional frequentist methods base on maximum-likelihood (MLE) or least-square estimation. We took the hierarchical Bayesian approach as a solution to the limited observations per experimental condition, considering that a large number of trial repetitions per condition (e.g., 60 trials) is typically required for frequentist modeling of ROC data.^34^ The power of hierarchical Bayesian modeling lies at the estimation of the population-level parameter by pooling information from lower level structures in data, such as subject-level and trial-level, while accounting for their variabilities simultaneously. Consequently, the individual parameter estimates can be informed by and shrunk toward the population-level posteriors^42^ and thus providing more robust fits. The advantage of the HBM is especially useful when a given dataset is limited by the number of subjects and/or trials per subject and condition, which is often the case in research of medical image expertise due to limited testing time with the specialized subject population.^43^

We built the hierarchical Bayesian DTSD model consisting of multiple hierarchical layers that the data contains, including trials, within-subject conditions (i.e., image exposure duration), between-subject conditions (i.e., radiological experience), and population-level. The main effects and interaction effects were estimated in a general linear model, sampled from the normal distribution where the mean is the sum of the fixed and random effects and the variability term is the interaction across effects.^44^^,^^45^ We took a total of 20,000 samples after 20,000 warm-ups from four chains of Markov chain Monte Carlo sampling. We chose noninformative and reasonably informative priors following previous studies with similar modeling approach.^46^ Statistical inference was made based on the mean of the posterior parameter distribution and its 95% credible interval (highest density interval, ${\mathrm{HDI}}_{95\%}$) of the population-level parameters.^47^ The ${\mathrm{HDI}}_{95\%}$ is conceptually similar to the frequentist 95% confidence interval, representing the smallest interval of parameter values covered by 95% of the posterior density and can be used as the Bayesian estimate of the certainty or the strength of evidence.

#### 2.3.2 ROC simulations and parameter recovery with the hierarchical Bayesian DTSD

**Fig. 3:**
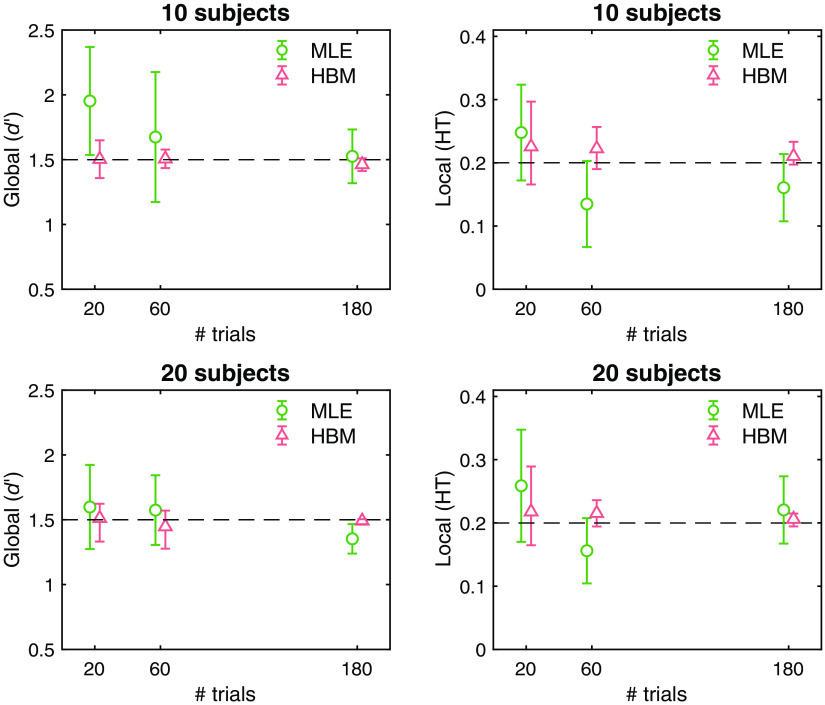
Recovery of the DTSD model parameters as a function of the number of subjects and trials, using fixed parameter values: global $(d^{\prime })=1.5$, and local (HT) = 0.2. The parameters were estimated under HBM and MLE methods for the same set of simulated ROCs. Green data points on this figure represent the means and the ${\mathrm{CI}}_{95\%}$ of the individual subject. Red data points represent the means and the ${\mathrm{HDI}}_{95\%}$ of the population-level parameter posteriors. The dashed-line represents the true parameter values used for data simulation.

To ensure the robustness of our HBM of the DTSD model over the traditional, nonhierarchical MLE method, we ran a set of simulations with known parameter values and then fitted the simulated data with both HBM and MLE methods. Specifically, each simulation generated data from the DTSD model function for every combination of $N_{\text{subjects}}=[10,20]$ and $N_{\text{trials}}=[20,60,180]$, with fixed parameter values of global $(d^{\prime })=1.5$ and local (HT) = 0.2. For HBM, we obtained a total of 12,000 samples, preceded by 12,000 warm-ups. Figure 3 summarizes the parameter recovery results by the HBM and MLE methods, as a function of $N_{\text{subjects}}$ and $N_{\text{trials}}$. The posterior mean and ${\mathrm{HDI}}_{95\%}$ of the estimated population-level parameters were quite robust (i.e., the means around the true parameter values with narrow ${\mathrm{HDI}}_{95\%}$) for HBM across the simulation sets but much less so for MLE, especially at the simulation sets with limited amount of data (e.g., $N_{\text{subjects}}=10$ and $N_{\text{trials}}=20$). These results can establish the robust parameter estimation of the hierarchical Bayesian DTSD model for the limited ROC dataset as this study.

As our primary interest is to capture the experimental effects (e.g., image exposure duration and radiological experience) on the DTSD model parameters, we ran another simulation focusing on the recovery of the condition effect. For simplicity, two ROC sets of hypothetical within-subject conditions were generated with reasonable preset DTSD parameter values based on the previous empirical estimates^5^^,^^21^ as follows:

$${\mathrm{cond}}_{A}:\;\text{global}(d^{\prime })=1.0,\;\text{local}(\mathrm{HT})=0.1,\;{\mathrm{cond}}_{B}:\;\text{global}(d^{\prime })=1.2,\;\text{local}(\mathrm{HT})=0.2,$$

where the condition effect (${\mathrm{cond}}_{B}-{\mathrm{cond}}_{A}$) is set to be, ΔHT=0.1, and $\Delta d^{\prime }=0.2$, meaning that performance for ${\mathrm{cond}}_{B}$ is better in both local and global components compared to ${\mathrm{cond}}_{A}$.

**Fig. 4:**
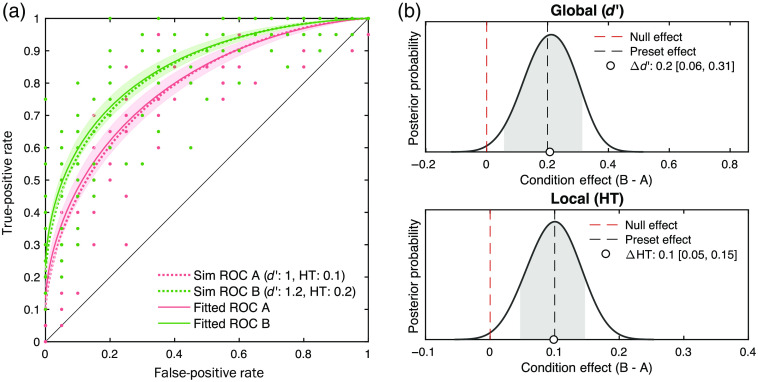
Recovery of the condition effect in the DTSD model parameters by the HBM method. ROCs were simulated separately for two hypothetical conditions: for condition A, global $(d^{\prime })=1.0$, local (HT) = 0.1; for condition B, global $(d^{\prime })=1.2$, local (HT) = 0.2. The condition effect (condition B to A) is thus defined as $\Delta d^{\prime }=0.2$ and ΔHT=0.1, respectively. Forty trials (20 target and 20 nontarget trials) for each of 13 subjects per condition were simulated. (a) The simulated (dotted-line) and (b) recovered (solid-line) ROC curves. The shaded error bars indicate the 95% ${\mathrm{CI}}_{95\%}$ of the recovered ROCs across subjects. Red and green colors represent condition A and B, respectively. Dots on this figure are simulated data points across subjects and confidence rating bins. (b) The recovered condition effect represented on the posterior probability functions of the group-level DTSD model parameters. The circle markers and the gray areas represent the means and the ${\mathrm{HDI}}_{95\%}$ of the population-level parameter posteriors (values shown in the legend). The black and red dashed-lines represent the preset condition effect and the null effect, respectively.

We generated 13 participants’ ROC data with 20 normal and 20 abnormal trials to mimic the data structure of this study. Figure 4(a) shows the simulated and recovered ROC curves. More importantly, Fig. 4(b) summarizes the recovered condition effect (${\mathrm{cond}}_{B}-{\mathrm{cond}}_{A}$) in the posterior probability of the group-level DTSD model parameters. First, the mean and ${\mathrm{HDI}}_{95\%}$ of both parameters were reliably centered around the true effects, $M(\Delta d^{\prime })=0.21$ [${\mathrm{HDI}}_{95\%}$: 0.06, 0.31], M(ΔHT)=0.10 [0.05, 0.15]. Second, the lower bound of ${\mathrm{HDI}}_{95\%}$ did not exceed over 0 (i.e., the null effect). Together, these parameter recovery tests demonstrate the benefits of hierarchical Bayesian modeling of ROC, over MLE methods, for its reliable parameter estimation for hypothesis testing, especially for datasets with a limited sample size and/or trial repetitions. In addition, these analyses suggest that the statistic power is sufficient for this study.

#### 2.3.3 Mouse trajectory analyses

Mouse trajectory data during the mammogram abnormality detection task were analyzed to assess the temporal dynamics of the global and local processes. On each trial and response, the trajectory data contained x−y cursor positions in pixel along the movement trajectory from the confidence scale onset to the final clicking response. We first corrected every x−y position to the relative distance from the cursor starting point (0, 0) at the center of the confidence scale. Second, the movement trajectory for each trial was normalized in time, from the movement onset to the final click response (0% to 100%). The movement onset was defined as the time when the cursor moved more than five pixels in any direction from the starting point.

Because our half-circle confidence scale was circular in nature, conventional distance-based measures on the raw Cartesian coordinates are at risk of artifacts, such as asynchronous variance in cursor positions across time and trials, which may in turn under- or overestimate the mid-flight trajectory deviations^46^ (e.g., change of mind). Instead, we focused on the moment-by-moment angular direction of cursor movements projected to the corresponding level of confidence on the confidence scale. We adopted a recently developed method of “destination-vector transformation^48^” that computes the progression of the mouse cursor movement based on two consecutive time points (from $t_{-1}$ to t), instead of the point-by-point angular positions at a given moment using a Cartesian-to-polar conversion. In other words, this analytic treatment detects where on the confidence scale a mouse cursor was heading toward at a given moment given its previous position (i.e., destination vector). Destination vectors thus can be considered as more accurate measures of internal action plan and its dynamic updating, especially during the mid-flight movements.

For each trial, we sampled cursor positions at every 5th normalized time points resulting in 21 data points from the starting point (0th) to the final click (100th), then computed 20 destination vectors (0% to 5%, 5th to 10th, … 95th to 100th). Instead of the summary statistics for point estimate of trajectory bias [e.g., area under curve (AUC), maximum deviation, or initial angle],^49^^,^^50^ we looked into the distributional property of mouse trajectory across trials, at each time point. Specifically, we examined whether or how the expertise effect (experienced–inexperienced) in detecting abnormality signal over noise (abnormal–normal) with different image exposure durations (second long to first short) emerged over the time course of the confidence rating mouse trajectory (i.e., three-way interaction effect).

## 3 Results

Both experienced and inexperienced groups showed above-chance (i.e., $d^{\prime }>0$) performance for the mammogram abnormality detection task, in both first and second responses with the image exposure durations of 500 ms and 2500 ms, respectively (short-first and long-second, hereafter). A two-way mixed-effect analysis of variance (ANOVA) for the task performance measured in $d^{\prime }$ revealed significant main effects of radiological experience (experienced versus inexperienced), F(1,24)=9.94, p=0.004, and $\eta_{p}^{2}=0.29$, and exposure duration (short-first versus long-second), F(1,24)=14.08, p=0.001, and $\eta_{p}^{2}=0.37$. More importantly, we found a significant interaction between the two factors, F(1,24)=10.68, p=0.002, and $\eta_{p}^{2}=0.32$, indicating that experienced participants showed greater improvement in their abnormality detection with longer image duration, as compared to inexperienced participants. In addition to $d^{\prime }$, we found similar results from another standard ROC measure of the AUC. The overall AUCs were above-chance (>0.50). The same two-way mixed-effect ANOVA for AUC again revealed significant main effects of radiological experience (experienced versus inexperienced), F(1,24)=8.21, p=0.009, and $\eta_{p}^{2}=0.26$, and exposure duration (short-first versus long-second), F(1,24)=9.26, p=0.006, and $\eta_{p}^{2}=0.28$. However, the interaction between the two factors did not reach a statistical significance, F(1,24)=3.02, p=0.095, and $\eta_{p}^{2}=0.11$.

### 3.1 Hierarchical Bayesian DTSD Modeling of ROC

**Fig. 5:**
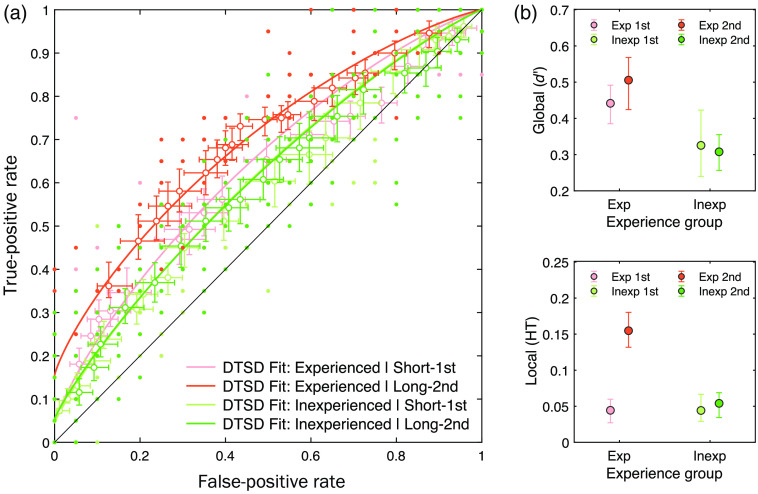
(a) Observed ROCs as a function of radiological experience (experienced versus inexperienced) and image exposure duration (short-first versus long-second), overlapped with the hierarchical Bayesian DTSD model fits (solid lines). The horizontal and vertical error bars indicate standard errors of false alarm (FA) rates and hit rates, respectively. The small dots indicate individual data points across participants and 19-bins confidence ratings. (b) The means and the ${\mathrm{HDI}}_{95\%}$ of posteriors of the population-level hierarchical Bayesian DTSD model parameters, global ($d^{\prime }$) and local (HT), as a function of experience group and image exposure duration.

The overall pattern can be identified in the ROCs [Fig. 5(a)], especially in that ROCs for the long-second responses from the experienced group were the most further away from the chance-level diagonal line as well as from the other conditions. To verify whether or how the expertise effect manifests in dissociable processes (i.e., continuous-global and discrete-local) in response to image duration (i.e., short-first and long-second), the raw ROCs were fitted with the hierarchical Bayesian DTSD model.

It is worth noting again that our hypothesis of dissociable cognitive processes makes distinct predictions for the manifestation of the radiological experience effect in mammogram abnormality detection. Specifically, for the short-first condition, the experienced participants would benefit more from their continuous-global processing of the initial gist of abnormality compared to the inexperienced participants. For the long-second condition, on the other hand, the radiological experience effect will primarily manifest in discrete-local processing of the focal lesion-based abnormality. The DTSD model is proposed to test this time-varying joint contribution of the global ($d^{\prime }$) and local (HT) processes in mammogram reading.

Figure 5(b) summarizes the means and ${\mathrm{HDIs}}_{95\%}$ of the posteriors of the group-level DTSD model parameters, as a function of radiological experience and image duration. The continuous-global component, global ($d^{\prime }$), showed a statistically credible effect in the comparison of experienced and inexperienced groups regardless of exposure duration (i.e., the main effect of radiological experience). Specifically, the experienced group showed an overall global ($d^{\prime }$), averaged between short-first and long-second, of 0.47 [${\mathrm{HDI}}_{95\%}$: 0.44, 0.51], whereas the inexperienced group had 0.32 [${\mathrm{HDI}}_{95\%}$: 0.27, 0.36]. The mean difference (i.e., experienced–inexperienced), 0.16 [${\mathrm{HDI}}_{95\%}$: 0.08, 0.23], was statistically credible, with the lower bound not crossing over zero (i.e., the null effect). All other effects (e.g., the effect of exposure duration or two-way interaction) were not statistically credible, including the within-experienced group comparison between short-first and long-second conditions, 0.06 [${\mathrm{HDI}}_{95\%}$: −0.05, 0.14].

The discrete-local component (local HT), in contrast, showed a distinctive pattern in that the experienced group, but not the inexperience group, showed increased local (HT) from the short-first to long-second responses. This pattern was confirmed by a credible two-way interaction effect, 0.10 [${\mathrm{HDI}}_{95\%}$: 0.04, 0.15], which is primarily driven by a credible difference (long-second to short-first) within the experienced group, 0.11 [${\mathrm{HDI}}_{95\%}$: 0.08, 0.14].

To summarize, our findings suggest a time-varying dissociation between global and local processes. Specifically, for the first report after brief image exposure, the better mammogram abnormality detection in experienced participants over inexperienced participants mainly manifested as the continuous-global processing, captured by the SDT component, global ($d^{\prime }$). For the second report with a longer presentation, only the experienced group participants showed improved performance from the first report. This effect was largely driven by the increase in the discrete-local processing captured by the high-threshold component, local (HT). In contrast, the strength of the SDT component did not further increase between responses in experienced participants, suggesting that global processing was saturated by 500 ms in the first response.

### 3.2 Unfolding Temporal Dynamics of Abnormality Detection from Mouse Trajectory

We hypothesized that continuous-global and discrete-local processes jointly attribute to radiological experts’ detection of mammogram. A major prediction for the dissociation between these processes includes a temporal scale, specifically the holistic perceptual gist can be acquired rapidly from the image onset, whereas the focal identification of malignant lesions would slowly emerge and potentially operate in parallel with the global process.^29^^,^^30^ In the aforementioned DTSD modeling of the empirical ROCs, we demonstrated the temporal dissociation by the experimental manipulation of image exposure duration. Next we used a model-free analytic approach to assess the mouse trajectory pattern during confidence rating for each response. This approach allows more precise investigation of temporal dynamics in global and local processes and how they influence the moment-by moment diagnostic decision making.

**Fig. 6:**
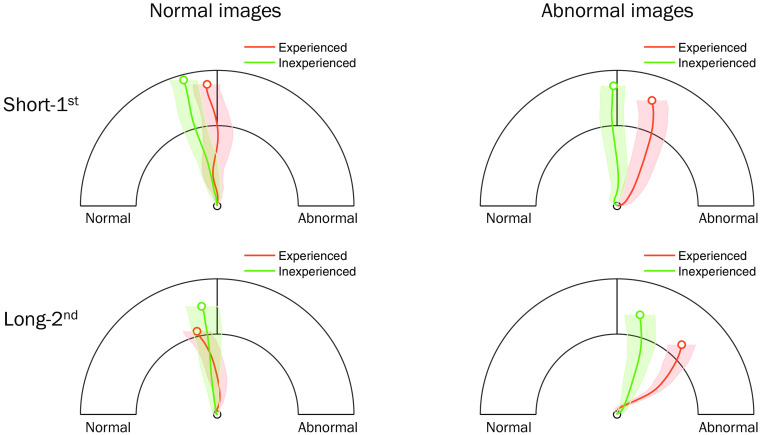
Mean mouse trajectories on a half-circle confidence rating scale as a function of radiological experience group (experienced versus inexperienced), pathology (normal versus abnormal), and image exposure duration (short-first versus long-second). From the central line on the scale, counterclockwise and clockwise areas correspond to normal and abnormal ratings along the continuous confidence scale from guess, probably, and sure. Error bars represent standard error of means for horizontal mouse cursor positions from movement onset to final click.

First of all, Fig. 6 illustrates the raw mouse trajectories on a half-circle confidence rating scale as a function of radiological experience group (experienced versus inexperienced), pathology (normal versus abnormal), and image exposure duration (short-first versus long-second). The patterns of confidence rating trajectory show that experienced participants were overall better in detecting abnormality from the images, and moreover, had greater gain in confidence toward a correct diagnostic decision with the extended viewing time (i.e., from short-first to long-second).

These continuous trajectory patterns are proposed to convey the temporal dynamics of the global and local processes and their unique contribution to mammogram abnormality detection that varied across time. To precisely assess this effect, we converted the raw mouse positions into the destination vectors that are assumed to represent the temporal unfolding of abnormality discrimination on the fly throughout the movement trajectory (see Sec. 2.3.3 for detail).

**Fig. 7:**
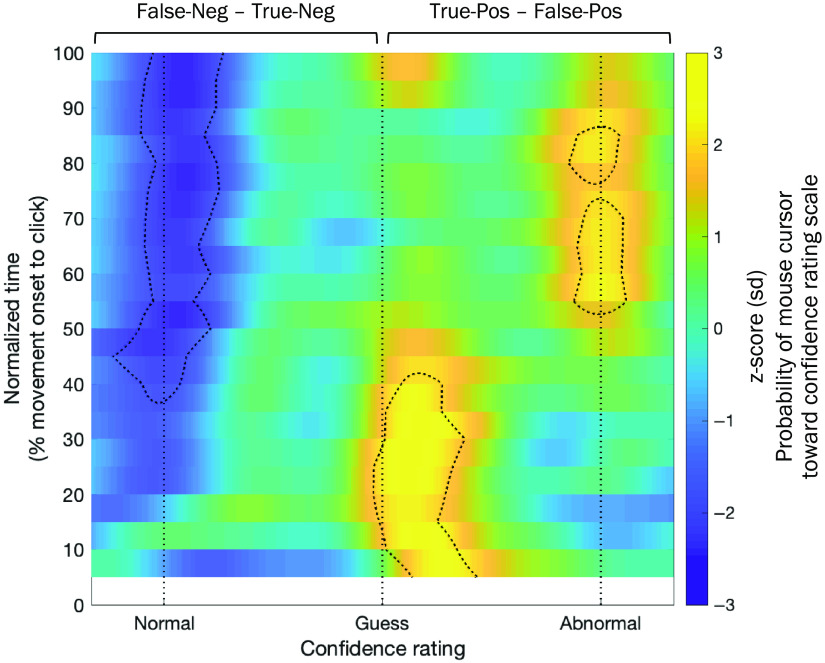
A 2D histogram depicting standardized group-aggregated mouse trajectory destination-vector distributions as a function of confidence rating (x axis), normalized time (y axis), and z-scored probability (z axis). A three-way interaction effect in mouse trajectory data: radiological experience group × pathology × image exposure duration, illustrating how the radiological expertise effect (experienced–inexperienced) in detecting the abnormality signal over noise (abnormal–normal) emerged differently between short- and long-image exposure durations (long-second to short-first). Since the effect plotted includes the difference between pathology condition (i.e., abnormal to normal), the probabilities on the right side from the midline indicate “hit rate–FA rate,” whereas the left side from the midline indicate “miss rate–correct rejection (CR) rate,” respectively. The dotted-contours on the histogram denote the areas of probability exceeding ±1.96 z-score (sd) of the three-way interaction effect.

**Fig. 8:**
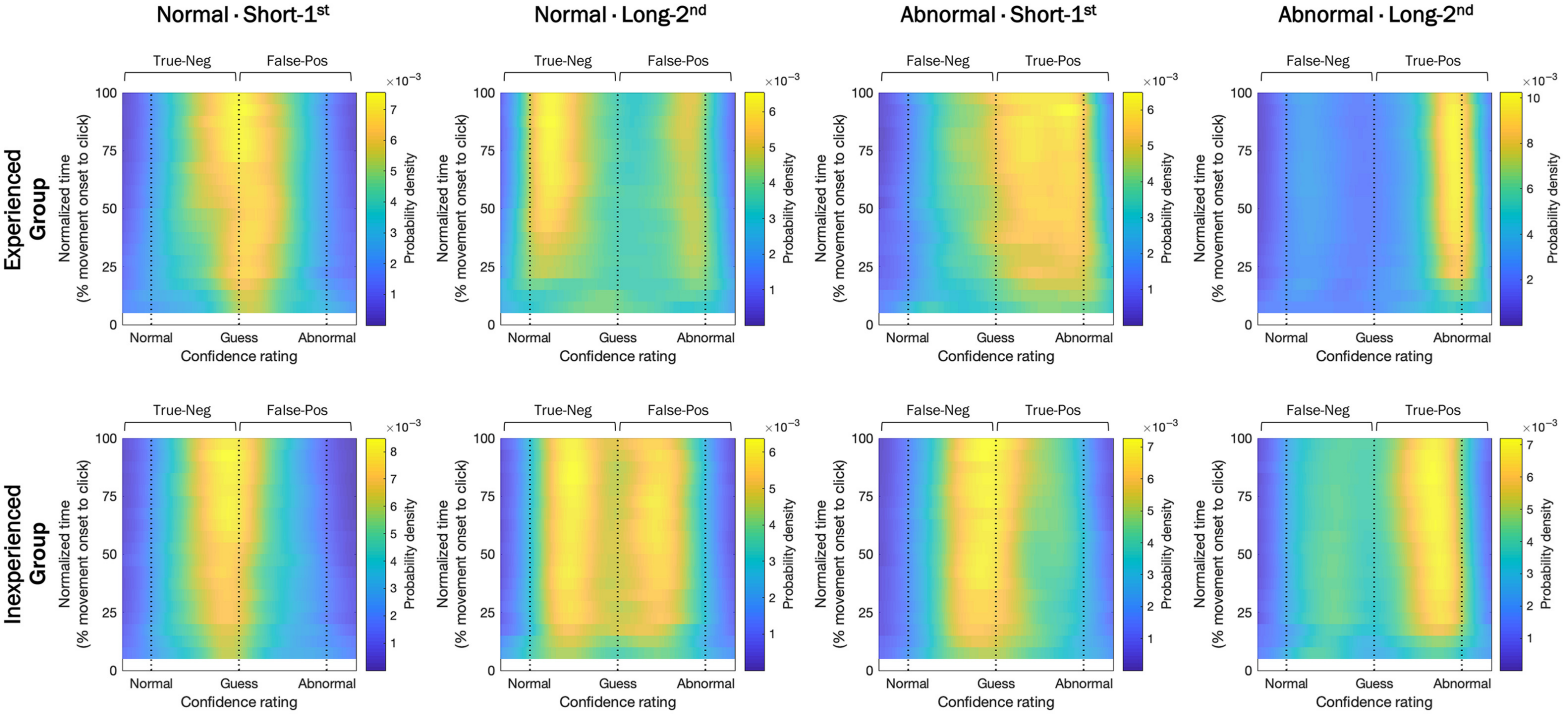
Aggregated raw mouse trajectory destination-vector distributions for each combination of radiological experience (experienced versus inexperienced), pathology (normal versus abnormal), and image exposure duration conditions (short-first versus long-second). For the normal image conditions, the probabilities on the left (toward normal) and right side (toward abnormal) of the midline indicate CR and FA responses, respectively. For the abnormal image conditions, the probabilities on the left and right side of the midline indicate miss and hit responses, respectively.

Of the primary interest, we focused on the three-way interaction effect, specifically how the radiological expertise effect (experienced–inexperienced) in detecting the abnormality signal over noise (abnormal–normal) emerged differently between short- and long-image exposure durations (long-second to short-first). The primary findings from the destination-vector trajectory distributions^46^ are shown in Fig. 7. The three-way interaction effect in aggregated trajectory data showed a distinctive pattern throughout the time course of mouse cursor movement trajectory [Fig. 7, also see the Appendix (Fig. 8) for the destination vectors for each condition]. Relative to the inexperienced group, the experienced group showed overall better identification of abnormality (true-positive–false-positive, yellow patches in Fig. 7) and better rejection of normal images (negative values of false-negative–true-negative, blue patches in Fig. 7) from the second response to the first response. Interestingly, this overall expertise effect manifested dominantly at the low-level confidence ratings (i.e., “guess” and “probably” abnormal responses) in the first ∼50% mouse movement trajectory. Immediately after, the effect rapidly shifted toward high-confidence ratings, leading to final clicking responses that were either confident identification of malignancy [z (true-positive–false-positive) >1.96; Fig. 7 top-right] or confident rejection of benign lesions [z (false-negative–true-negative) <−1.96; Fig. 7 top-left]. Interestingly, this temporal dynamic is consistent with the initial automatic process (e.g., coarse grained information and familiarity) with lower confidence and the later controlled process (e.g., fine-grained information and recollection) with high confidence in perception^8^ and recognition memory.^51^^,^^52^ It is thus likely the early low-confidence and later high-confidence ratings in reporting mammogram abnormality are driven by global and local information processing, respectively. Together, these results provide further support for the dissociation between continuous-global and discrete-local processing in accounting for the expertise effect in mammogram abnormality detection.

## 4 Discussion

This study developed a model-based approach to assess the contributions and the time course of continuous-global and discrete-local processing in mammogram abnormality detection. We hypothesized these two dissociable visual processes of mammogram images jointly contribute to mammogram expertise but with different weights and temporal dynamics. To test this hypothesis, we modeled experienced versus inexperienced observers’ performance (ROC) at first (500 ms) versus second (2500 ms) mammogram image presentation in a mammogram abnormality detection task with the DTSD model. For the initial short presentation of mammogram images, the experienced participants were better at detecting the global gist of abnormality, manifested as higher SDT component, global ($d^{\prime }$). For the second presentation of the same mammogram images for the longer duration, the experienced participants, but not the inexperienced participants, showed increased task performance that was largely driven by better processing of local information, manifested as higher local (HT) component, whereas the global processing of abnormality, global ($d^{\prime }$), remained saturated from the first exposure. This suggests that experienced participants, when given enough time, were able to examine local parts of the image and identify a focal sign of malignant lesions.

Second, we assessed the temporal dynamics of mammogram abnormality detection by modeling mouse movement trajectory of the confidence rating responses. We purposefully looked into the three-way interaction effect, showing how experienced participants discriminated abnormal images from normal images and how these differed between short- and longer-image exposure durations. We identified an interesting trajectory pattern in this interaction effect. Specifically, from the first reading to second reading, the experienced observers, as compared to inexperienced observers, initially reported image abnormality with low confidence, presumably based on by global gist-based processes, and then rapidly shifted to high-confidence discrimination of image abnormality, presumably driven by identification, and rejection of focal signs of malignancy.

Together, these results not only demonstrate that continuous-global and discrete-local processing of mammogram images could be analytically dissociated and modeled but also provide empirical evidence supporting our hypothesis that these processes, with different weights and time courses, can jointly account for the expertise effect in medical image perception. This study can thus make some empirical, theoretical, and methodological contributions to medical image perception research in the following aspects. First, although the influential holistic processing hypothesis is a popular account of visual expertise, it is unclear whether and how it applies to medical expertise in breast cancer imaging. This study has obtained preliminary evidence that mammogram expertise (experienced versus inexperienced observers) manifests largely in holistic processing but only at the early phase of mammogram reading. More confident decisions occur afterward based on the later local processing. Second, this study has developed a model-based approach (the ROC mixture model, DTSD) to quantitatively isolate and estimate global and local processing without artificial experimental manipulations. Third, the HBM significantly reduces the number of trials (and hence testing time) that is needed for reliable parameter estimation, as compared to the traditional model fitting methods. Finally, the analyses of mouse trajectory, an easily available measure, can recover the temporal dynamics of the diagnostic reading without special research equipment like eye trackers. These methodological advantages make our approach more practical and ecologically valid in breast cancer imaging research.

Note that our DTSD model is analytically and also conceptually consistent with the DPSD models in the recognition memory literature. The DPSD models propose that recognition judgments in long-term memory^37^ as well as short-term memory^35^^,^^53^ can be attributed jointly by recollection and familiarity that are independent each other in mnemonic contents and/or processes. The present findings support the dissociation between the global gist and the focal identification of mammogram abnormality and their time courses could be, at least in part, attributed to processes related to familiarity and recollection, respectively, for the following reasons. First, recollection and familiarity are assumed to be driven by local (e.g., item) and global (e.g., associative) information and measured as the HT and SDT components of empirical ROCs, respectively. In fact, there is a number of empirical studies that established a characterization of global configural processing as familiarity and the SDT component (e.g., global matching signal^54^) and discrete item-based as recollection and the HT component.^35^^,^^55^^–^^57^ The SDT and HT components may also capture subconscious “sensing” and conscious “seeing” in perception,^38^ respectively. Second, the different temporal dynamics of the two processes in this study are also consistent with the mounting evidence suggesting that the $d^{\prime }$ measure is sensitive to phenomenal, subliminal, and faster decisions (e.g., “feeling of knowing”), in contrast to explicit and slower recollection process.^51^^,^^52^^,^^58^^,^^59^

Several caveats of the current study entail further investigation. First, the lack of additional increase in the global ($d^{\prime }$) parameter from the short to the longer exposure duration in the experienced observers suggest that the global holistic processing may have saturated after the initial glimpse of the image. This is inconsistent with some previous ideas that holistic processing can continue throughout the trial in parallel or by alternating with focal identification of the sign of abnormality.^14^^,^^60^^,^^61^ However, given our manipulation of image presentation duration for 500 and 2500 ms, it is possible that experts’ holistic processing of the image would have already been completed within the first presentation then they primarily focused on localizing the abnormal region in the image during the second presentation that in turn led to the increased likelihood of discrete diagnosis of abnormality. Second, mouse trajectory analyses have previously been utilized in testing various cognitive processes^62^^–^^65^ and allowed precise investigation of the temporal dynamics of underlying processes during decision-making in this study. However, it does not provide direct evidence for the latent cognitive representations and processes. Future research needs to adopt selective experimental manipulations of global versus local processing^5^ in testing mouse trajectories. Third, the computational principles and precise representation for global information in medical images is not as well defined as those in vision research.^5^^,^^66^^,^^67^ Better understanding of this aspect would be critical for research in medical image perception. Finally, this study was conducted in a conference setting and had various restrictions in recruiting participants with specific radiological experience and specialty as well as time allowed for volunteered experiment participation. Although we leveraged the limited number of datapoints (e.g., total of 40 cases reading) to shed light on the utility of HBM in ROC analysis, further replication would be needed to confirm the current findings with a proper control of expertise, reliable number of trials, and realistic image reading environment.

Understanding the nature of medical expertise in breast cancer imaging could be pivotal for cancer diagnostics, clinical training, and the development of computer-aided detection programs. This project aims to directly assess the mental black box (i.e., underlying cognitive mechanisms) of medical expertise in breast cancer imaging. Future research is needed to link the model-based assessment of global processing in mammogram reading to acquisition of mammogram expertise and its potential neural substrate (i.e., FFA)^68^ and generalize the present findings to different domains of cancer imaging (e.g., colonoscopy).

## 5 Appendix

The aggregated raw mouse trajectory destination-vector distributions for each combination of radiological experience, pathology, and image exposure duration conditions are shown in Fig. 8.
